# Renal cell carcinoma with fibromyomatous stroma (RCC FMS) and with hemangioblastoma‐like areas is part of the RCC FMS spectrum in patients with tuberous sclerosis complex

**DOI:** 10.1111/his.15505

**Published:** 2025-07-01

**Authors:** Katherina Baranova, Jacob A Houpt, Deaglan Arnold, Andrew A House, Laura Lockau, Lindsay Ninivirta, Stephen Pautler, Haiying Chen, Madeleine Moussa, Rola Saleeb, Jose A Gomez, Asli Yilmaz, Farshid Siadat, Adrian Box, Douglas J Mahoney, Franz J Zemp, Manal Gabril, Kiril Trpkov

**Affiliations:** ^1^ Department of Pathology and Laboratory Medicine London Health Sciences Centre London ON Canada; ^2^ Schulich School of Medicine and Dentistry, London Health Sciences Centre London ON Canada; ^3^ Department of Medicine Western University and London Health Sciences Centre London ON Canada; ^4^ Divisions of Urology and Surgical Oncology, Departments of Surgery and Oncology Western University London ON Canada; ^5^ Department of Laboratory Medicine and Pathobiology University of Toronto Toronto ON Canada; ^6^ Diagnostic and Molecular Pathology Cumming School of Medicine, University of Calgary Calgary AB Canada; ^7^ Department of Biochemistry and Molecular Biology, Arnie Charbonneau Cancer Institute, Alberta Children's Hospital Research Institute Cumming School of Medicine, University of Calgary Calgary AB Canada

**Keywords:** clear cell, fibromyomatous stroma, haemagioblastoma, kidney, leiomyomatous stroma, renal cell carcinoma (RCC), TSC

## Abstract

**Aims:**

Sporadic renal cell carcinomas with fibromyomatous stroma (RCC FMS) with coexistent haemangioblastoma (HB)‐like morphology have been previously reported. Such morphology has not, however, been documented in patients with tuberous sclerosis complex (TSC). We evaluated clinicopathologic, immunohistochemical and molecular findings of RCC FMS with HB‐like features in three TSC patients.

**Methods and results:**

All three patients were females with confirmed germline mutations in *TSC1* in the absence of *VHL* or other alterations. One patient had bilateral nephrectomy with six separate tumours (three in each kidney), and two patients had one tumour each, resected by partial nephrectomy. Prominent fibromuscular stroma was present at the tumoral periphery and surrounding clear cell nests forming tubules and papillary formations (RCC FMS areas), admixed with solid, clear to eosinophilic and spindle cell areas (HB‐like areas). HB‐like areas were variably represented in individual tumours, but in some, HB‐like features were almost exclusive. HB‐like areas were negative for CK7 and showed inhibin‐α and S100 reactivity, in contrast to typical RCC FMS areas, which were CK7 positive, while negative for inhibin‐α and S100. GPNMB, which is consistently expressed in *TSC/mTOR* altered tumours, was positive in both components. On follow‐up (10 months–21 years), all patients had an indolent course.

**Conclusions:**

HB‐like morphology found in RCC FMS in TSC patients represents part of the morphologic spectrum of RCC FMS, which is a previously underreported and underrecognized feature. RCC FMS with HB‐like areas show uniform GPNMB reactivity and are associated with *TSC/MTOR* alterations, but not with *VHL* alterations.

## Graphical abstract

We report on three patients with Tuberous Sclerosis Complex (TSC) who had renal cell carcinomas with fibromyomatous stroma (RCC FMS), demonstrating a coexistent haemangioblastoma (HB)‐like morphology. Our findings support the conclusion that HB‐like features are part of the spectrum of RCC FMS with *TSC/MTOR* alterations.

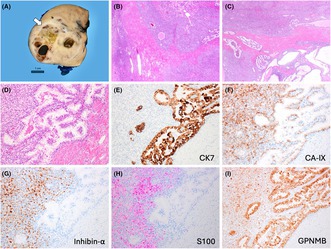

## Introduction

Renal cell carcinoma with fibromyomatous stroma (RCC FMS) has been proposed as a novel renal entity by the Genitourinary Pathology Society (GUPS) in 2021.^1^ However, this entity has been previously described under different names that emphasized the presence of smooth muscle/leiomyomatous or fibromatous stromal component, such as: mixed renal tumour with carcinomatous and fibroleiomyomatous components,^2^ RCC associated with prominent angioleiomyoma‐like proliferation,^3^ clear cell RCC with smooth muscle stroma,^4^ RCC with clear cells, smooth muscle stroma and negativity for 3p deletion,^5^ RCC with leiomyomatous stroma^6^ and RCC with angioleiomyomatous‐like stroma^7^ (also reviewed in Trpkov and Hes^8^). Although this entity was included as an ‘emerging/provisional’ entity in the 2016 World Health Organization (WHO) Classification of Tumours of the Urinary System as ‘RCC with (angio)leiomyomatous stroma’,^9^ it has not been included in the 2022 WHO classification.^10^

RCC FMS is characterized by an epithelial component of cells with clear cytoplasm forming larger nests of compact branching tubules and papillary formations, set in a fibromuscular stromal background, typically more prominent at the periphery and variably intersecting the epithelial nests.^1^, ^11^, ^12^ On immunohistochemistry (IHC), the epithelial cells are positive for CK7, CAIX, as well as for CD10 and vimentin.^1^, ^11^, ^12^ RCC FMS may arise in a sporadic setting^11^ or, less frequently, in a hereditary setting in patients with tuberous sclerosis complex (TSC)^13^, ^14^ and in both scenarios, there is usually an association with pathogenic variants in *TSC1* or *TSC2* in the mTOR signalling pathway. Generally, renal tumours occur in about 2%–4% of patients with TSC and such *TSC/mTOR* altered tumours, in addition to RCC FMS morphology, may demonstrate other distinct morphologies.^13^, ^14^, ^15^ Importantly, in both hereditary and sporadic settings, RCC FMS frequently harbours mutations in *TSC1*, *TSC2* and *MTOR* ^1^ typically in the absence of *VHL* alterations, including absence of hypermethylation and loss of heterozygosity at 3p.

A recent study by Kojima *et al*.^16^ described four examples of ‘RCC with hemangioblastoma (HB)‐like features’ in a sporadic setting (i.e. in patients without TSC) that, in our view, had histologic, IHC and molecular similarities to RCC FMS, and they proposed that this entity was distinct from clear cell RCC. Notably, 2/4 cases demonstrated *MTOR* mutations, but no *VHL* alterations were found.^16^ The HB‐like morphology was composed of cells with clear and eosinophilic cytoplasm, with delicate vasculature in the background, resembling HBs of the central nervous system and ‘peripheral HBs’ (also described as ‘extraneural/extraneuraxial’ HBs), including those in the kidney.^17^, ^18^, ^19^, ^20^, ^21^, ^22^, ^23^, ^24^ Although RCC FMS is well‐documented in a TSC setting,^13^, ^14^, ^15^ to our knowledge, HB‐like morphology has not been previously reported in RCC FMS in TSC patients. In this study, we report three patients with known TSC who had RCC FMS with admixed HB‐like features. The morphologic, IHC and molecular features of these tumours, in our opinion, are very similar to those reported in sporadic RCC FMS with HB‐like features. It is currently unclear whether such a constellation of findings represents a distinct entity, or if RCC FMS with HB‐like areas belongs to the spectrum of RCC FMS.

## Materials and Methods

### Patient Selection

We identified three patients with TSC who had renal tumours that showed RCC FMS with HB‐like areas, out of a cohort of over 75 adult patients with TSC, followed in the institutional clinic (by one of co‐authors AAH). After encountering patient 1 and patient 2 in routine practice and performing consultations with expert uropathologists, we retrieved the third case (patient 3) from the institutional files of London Health Sciences Centre, ON, Canada. We obtained individual informed consent from all three patients, and we followed the tenets of the Declaration of Helsinki in conducting the study.

### Immunohistochemistry

We performed IHC staining on Autostainer Link 48 platform using the EnVision Flex System (Agilent Technologies) using a heat‐based antigen retrieval method and high‐pH buffer. On‐slide multi‐tissue positive and negative controls were used in all cases to confirm adequate IHC staining. We used the following antibodies for IHC work‐up in all cases: CK7 (OVTL 12/30; Dako GA619; prediluted ready to use; Dako, CA, USA), CAIX (NovocastraTM Liquid Mouse Monoclonal Antibody; 1:100), S100 (Polyclonal, Dako GA504; prediluted ready to use; Dako, CA, USA), Inhibin‐α (R1; Dako GA058; prediluted ready to use; Dako, CA, USA), CD10 (DAK‐CD10; Dako GA786; prediluted ready to use; Dako, CA, USA), vimentin (V9; Dako GA630; prediluted ready to use; Dako, CA, USA) and AE1/AE3 (Dako IR053; prediluted ready to use; Dako, CA, USA); additional IHC stains were also done in individual cases. We also performed IHC staining for glycoprotein non‐metastatic melanoma protein B (GPNMB) IHC using a rabbit monoclonal antibody directed against human GPNMB protein residues near the N‐terminus (clone E4D7P; 1:1000 dilution; Cell Signaling Technology), following published protocols.^25^, ^26^ IHC stains were scored as: positive (>50% cells) and focal positive (<50% of cells).

### Targeted DNA Next Generation Sequencing

We performed a comprehensive Cancer Biomarker DNA Panel at the University of Calgary, using an established institutional protocol. Cancer Biomarker Comprehensive DNA panel is a pan‐solid tumour assay using next generation sequencing (NGS) validated to detect single nucleotide variants (SNV), small insertions and deletions (indels), amplifications and homozygous‐type deletions using a panel of 130 genes including *TSC1*, *TSC2*, *MTOR*, *ELOC* and *VHL* (full list of genes in the panel and additional NGS information are available in the Supplemental Data ‐ NGS Methods). In short, DNA was extracted from formalin fixed, paraffin embedded (FFPE) block from specimens, followed by template preparation and massively parallel sequencing using the Ion Chef System and Ion GeneStudio S5 Systems. Base calling was generated by the Torrent Suite Software (version 5.18.1). Variant calling, Oncomine variant annotations and tumour mutational burden were generated by Ion Reporter software (version 5.16) with alignment to the reference human genome GRCh37/hg19.

## Results

Clinicopathologic and molecular findings, as well as patient follow‐up information are summarized in Table 1.

**Table 1:** Clinicopathologic and molecular findings and follow‐up of three TSC patients with RCC FMS and HB‐like areas

Patient	Age/sex	Germline testing	Somatic testing	Surgery	Size (cm)	HB areas	F/U
1	19 F	Copy number variant, deletion of 9q34.13q34.2, with loss of 6 OMIM morbid genes: *TSC1*, *MED27*, *NTNG2*, *SETX*, *GFI1B*, *CEL*	Not performed	Laparoscopic robot assisted partial nephrectomy	3.5	70%	12 mo, NED
2	27 F	*De novo TSC1* frameshift resulting in stop codon truncation. c.965dupT (p.Met322Ilefs*19)	Same mutation: *TSC1* p.Met 322Ilefs*19	Bilateral nephrectomy and transplant	1–2.1 (× 3 left) 0.4–1.3 (×3 right)	10%–100% (left) 20%–90% (right)	10 mo, NED
3	17 F	*De novo TSC1* two base pair deletion: frameshift. c.1781_1782del	*TSC1* p.Val594 GlyfsTer11 and *TSC1* p.Lys 890Ter	Partial nephrectomy	2.5	5%	21 y, NED

F, female; NED, no evidence of disease.

### Clinicopathologic Features

Patients were 17–27 years old Caucasian females, all clinically demonstrating TSC manifestations at an early age. All patients presented with asymptomatic renal masses on routine imaging. Patient 2 had bilateral nephrectomy to remove 6 tumours (3 in each kidney, with additional, separate 0.3 cm angiomyolipoma in the left kidney), followed by a living donor kidney transplant, because of progressively declining renal function and a prior biopsy‐proven RCC. The remaining two patients (#1 and #3) had one tumour each, resected by partial nephrectomy. All tumours were relatively small (0.4–3.5 cm), stage pT1a, and all patients had indolent clinical course. In one patient (#3) the follow‐up was 21 years.

**Figure 1:**
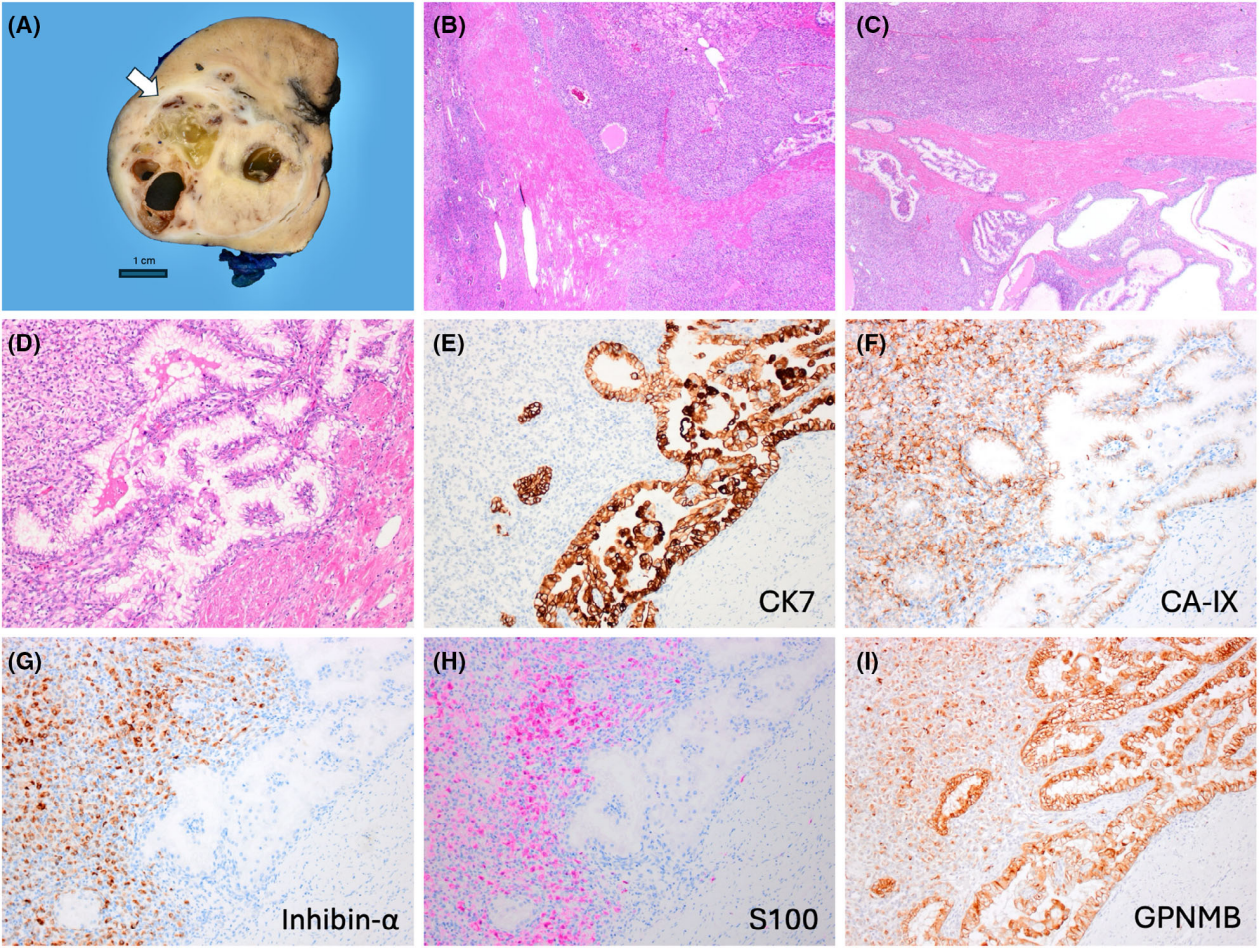
Patient 1, RCC FMS with predominant HB‐like area. (**A**) Grossly, the tumour is circumscribed, grey to light brown with a recognizable stromal band at the periphery (arrow). (**B**) At low power, the fibromuscular stroma is prominent at the periphery, and the tumour appeared mostly solid. (**C**) The inner part of the tumour showed two admixed morphologies: solid HB‐like (top and lower left) and RCC FMS clear cell areas forming nests, tubulocystic and papillary formations. (**D**) The three components are illustrated at high power: HB‐like (upper left), RCC FMS with clear cells (center) and stroma (lower right). (**E**–**I**) The IHC area illustrated in (**D**) shows a different immunoprofile in the HB‐like and RCC FMS clear cell areas. The clear cell area is positive for CK7, while the HB‐like area is negative (**E**); both areas are positive for CAIX (**F**); Inhibin‐α and S100 are positive in HB‐like areas, while the clear cell area is negative for both (**G**–**H**); GPNMB is positive in both areas (**I**). Fibromuscular stromal area (lower right in **D**–**I**) is negative for all stains.

**Figure 2:**
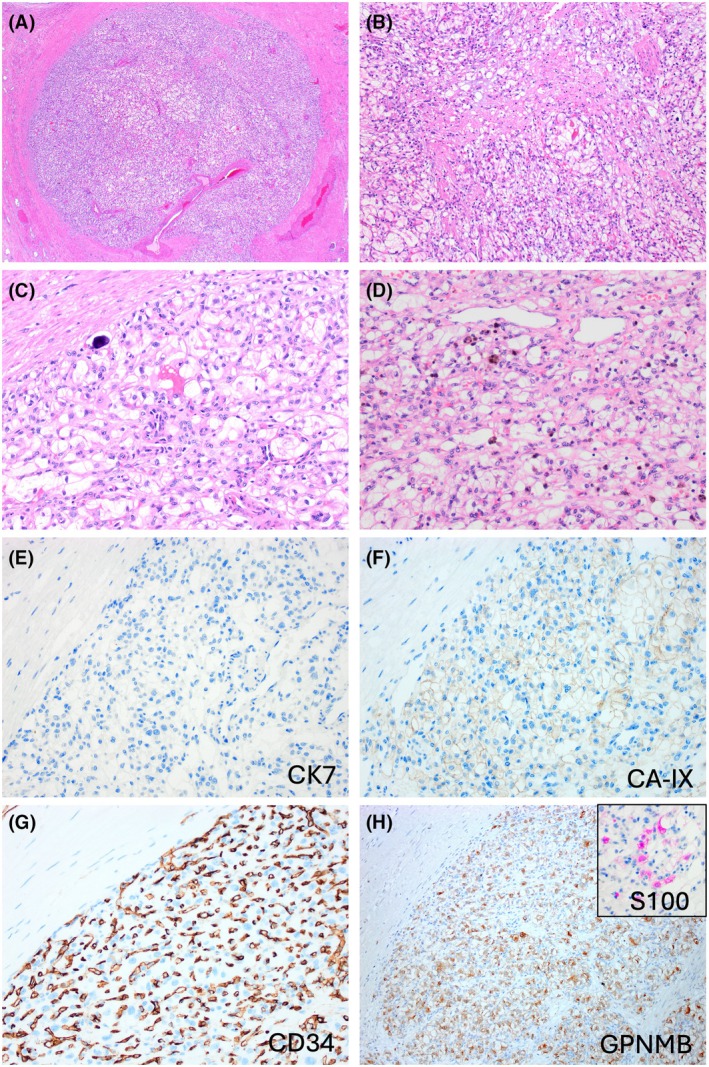
Patient 2 had bilateral nephrectomy to remove 6 tumours (3 in each kidney). (**A**) One tumour from the right kidney measured 0.5 cm. At low power, it showed an almost exclusive HB‐like morphology, with solid growth surrounded by a thick fibromuscular layer. (**B**) Stroma was focally admixed with the epithelial areas. (**C**, **D**) HB‐like areas were composed of epithelioid cells with mostly clear cytoplasm, interspersed with spindle stromal cells, focal lipoblast‐like cells, as well as rare psammoma bodies (**C**) and hemosiderin deposits (**D**). (**E**) CK7 was uniformly negative in HB‐like areas. (**F**) CAIX showed delicate membranous reactivity in HB‐like areas. (**G**) The delicate vascular architecture is highlighted by CD34. (**H**) GPNMB was uniformly positive and S100 was focally positive (inset).

**Figure 3:**
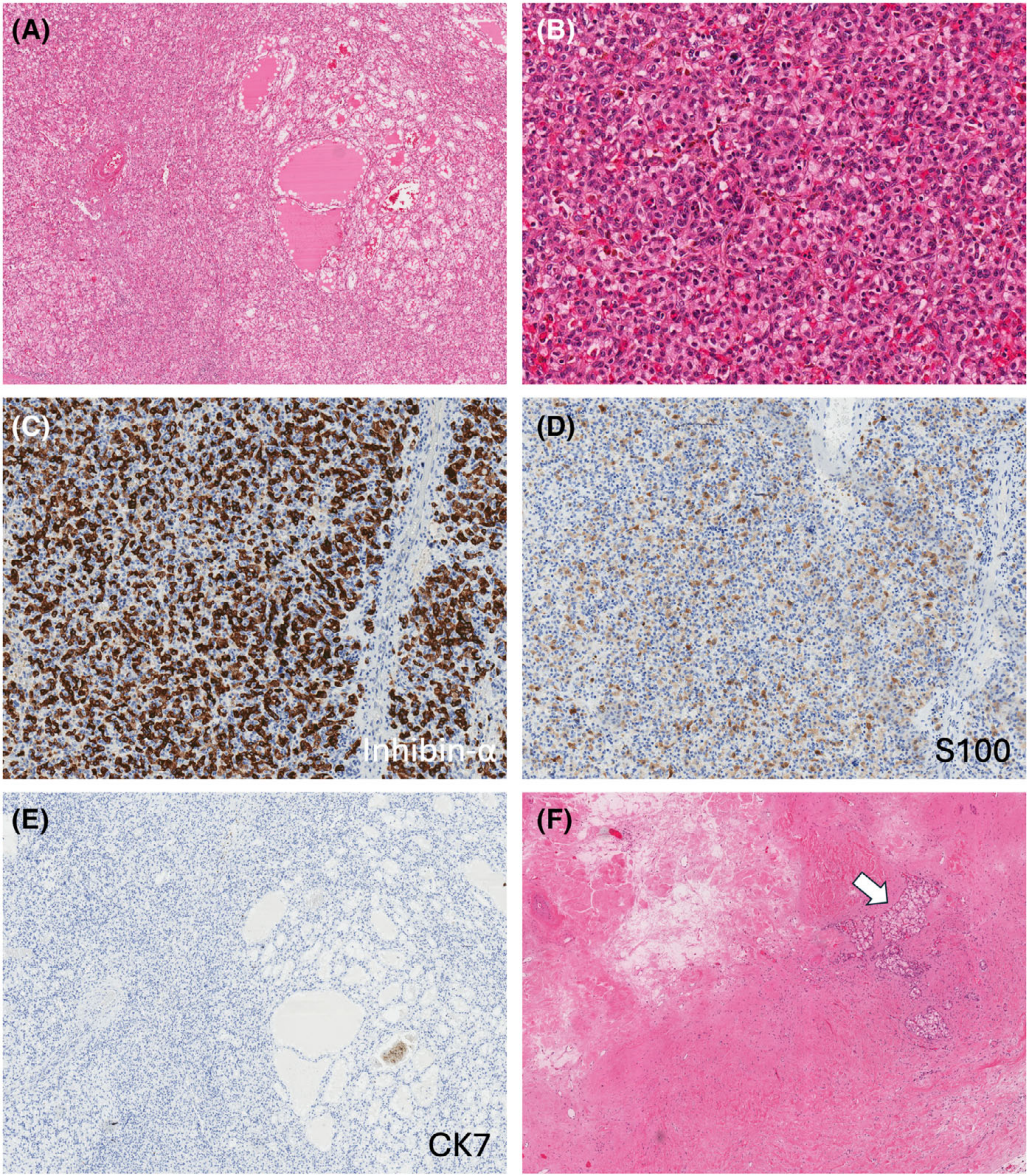
Patient 2. Another tumour from the left kidney that measured 1.8 cm is illustrated in **A**–**E**. (**A**) The tumour demonstrated predominantly solid HB‐like growth with only focal tubular and tubulocystic areas. (**B**) HB‐like areas exhibited solid growth and mostly eosinophilic cells, set in a delicate vascular background. (**C**, **D**) The tumour was diffusely positive for inhibin‐α (**C**) and focally for S100 (**D**). (**E**) CK7 was uniformly negative. (**F**) A separate tumour in the same kidney was almost completely hyalinized with only small recognizable clear cell nests (arrow).

**Figure 4:**
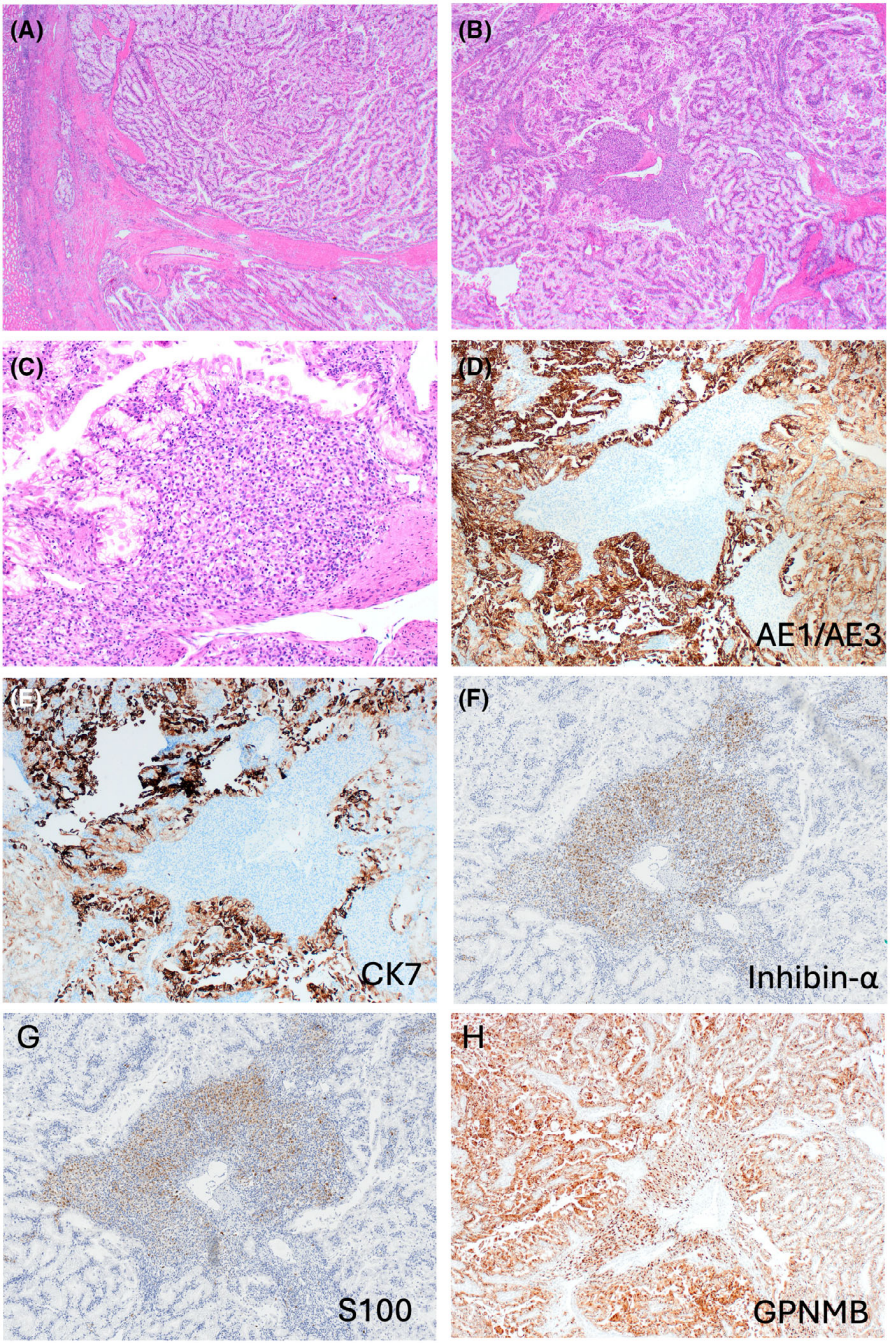
Patient 3 had RCC FMS with dominant clear cell areas and very focal HB‐like areas (~5%). (**A**–**C**) There was a thick fibromuscular stroma at the periphery that intersected the cellular areas (**A**), composed of clear cells that formed variable size nests of branching tubules, tubulocystic and papillary formations (**B**, **C**); a focal solid HB‐like area can be seen centrally (**B**, **C**). (**D**, **E**) Clear cell areas in RCC FMS were reactive for AE1/AE3 (**D**) and CK7 (**E**). (**F**, **G**) In contrast, clear cell areas were negative for inhibin‐α (**F**) and S100 (**G**), while the central HB‐like area was positive for both. (**H**) GPNMB was diffusely positive in both clear cell and HB‐like areas.

Grossly, all tumours were circumscribed, with grey to light brown cut surfaces and had a recognizable stromal band at the periphery (Figure 1A). The fibromuscular stroma invariably extended into the inner tumour parts. The tumours typically showed two admixed morphologies—RCC FMS‐like and HB‐like that were variably represented in all tumours (Figures 1, 2, 3, 4). RCC FMS areas were composed of clear cells with voluminous cytoplasm, forming variable‐sized nests of elongated, frequently branching tubules, tubulocystic and papillary formations (Figures 1B–D, 3A and 4A–C). HB‐like areas had uniformly solid growth with epithelioid cells showing eosinophilic to focally clear cytoplasm, interspersed with spindle stromal cells with focal atypia, as well as rare psammoma bodies and hemosiderin deposits (Figures 1B–D, 2A–D and 3A,B). A delicate capillary vasculature was invariably present in the background (Figure 3B). The extent of HB‐like areas in individual tumours varied from 5% to 100%. One tumour in patient 2 was almost completely hyalinized with only small residual clear cell nests (Figure 3F). Very focal coagulative necrosis (~5%) was found in one tumour (patient 1), but no other adverse findings were present in the remaining tumours. Of note, the initial biopsy performed in patient 1 showed only HB‐like tumour with exclusive solid growth (Figure S1A–F); on resection, the HB‐like areas were still dominant (~70%), but there were also recognizable RCC FMS areas (~30%) (Figure 1B–D).

### Immunohistochemistry Findings

On IHC, the HB‐like areas in all tumours were positive for inhibin‐α and S100 (in some tumours focally) but they were negative for CK7 and AE1/AE3 (Figures 1E–H, 2E, 3C–E and 4D–G). In contrast, typical RCC FMS clear cell areas were strongly and diffusely positive for CK7 and AE1/AE3 but were uniformly negative for inhibin‐α and S100 (Figures 1E–H, 4D–G). Both morphologies were positive for CAIX (Figures 1F and 2F), CD10, vimentin and GPNMB (Figures 1I, 2H, 4H). PAX8 was positive in both components, although HB‐like areas had more focal reactivity. IHC results for both components in all tumours are shown in Table 2. The fibromyomatous stromal (FMS) component was non‐reactive for all markers except for desmin and smooth muscle actin. Additional IHC performed in each tumour is detailed in Table S1.

**Table 2:** Immunohistochemical findings in RCC FMS with HB‐like areas comparing the HB‐like areas and the RCC FMS‐like clear cell areas

	Patient 1		Patient 2		Patient 3	
	HB‐like	RCC FMS‐like	HB‐like	RCC FMS‐like	HB‐like	RCC FMS‐like
CAIX	+	+	+	+	+	+
CK7	−	+	−	+	−	+
Inhibin‐α	+	−	+	−	+	−
S100	+	−	+	−	+	−
CD10	+	+	+ (foc)	+	+	+
Vimentin	+	+	+	+	+	+
GPNMB	+	+	+	+	+	+

### Molecular Findings

All patients had known pathogenic germline mutations in *TSC1*, as shown in Table 1. In two tumours (from patients 2 and 3), mutations were additionally confirmed on molecular analysis using a comprehensive Cancer Biomarker DNA Panel. Importantly, *VHL* mutations or loss of heterozygosity at 3p were not identified in any patient.

Patient 1 had a family history of a known pathogenic microdeletion and initially underwent a screen with single gene Multiplex Ligation dependent Probe Amplification, which utilized multiplex PCR to detect single gene *TSC1* deletion, in a context of known familial microdeletion. The original deletion was diagnosed in patient's immediate family member by targeted microarray assay, showing a pathogenic interstitial copy number loss of approximately 1.5 Mb of chromosome region 9q34.13q34.2 encompassing 6 OMIM Morbid genes including *TSC1*.

Patient 2 was clinically diagnosed with TSC and received genetic counselling and targeted genetic testing of *TSC1* and *TSC2* via blood draw, which identified one possible pathogenic variant in the T*SC1* gene c.965dupT (p.Met322Ilefs*19). Both parents also underwent genetic testing which confirmed that neither of them carried this variant and it was considered ‘de novo’.

Patient 3 was diagnosed with TSC in 2003 and full records of the methodology used to detect the variant were not available. The patient had molecular genetic testing which demonstrated a likely damaging sequence variant within the *TSC1* gene (c.1781_1782del), resulting in a 2 bp deletion causing a frame shift within the *TSC1* gene, predicted to disrupt the structure and function of the *TSC1* protein product (Hamartin). Both parents were not found to have the *TSC1* sequence variant, suggesting that it was ‘de novo’.

## Discussion

In this study, we report on RCC FMS with distinct HB‐like areas found in three patients with TSC. To our knowledge, this is a novel and not previously reported observation of such combined morphology in renal tumours of TSC patients. Both tumoral components were imperceptibly admixed in various proportions in individual tumours. Such variability and complex cellular architecture argue against the possibility that these two components belong to different (‘collision’) tumours but rather represent different aspects of one tumour type. Moreover, on IHC, although both components had distinct differences, they also showed some IHC similarities. The typical RCC FMS clear cell areas were uniformly and strongly positive for CK7 and AE1/AE3, CAIX, vimentin and for CD10,^1^, ^27^ but were negative for S100 and inhibin‐α. In contrast, the HB‐like areas were typically reactive for S100 and inhibin‐α but were uniformly negative for CK7. Both morphologies expressed CAIX, vimentin and CD10. In this study, we also used GPNMB, a transmembrane protein transcriptionally activated by *TFE3* and *TFEB*, which was proposed as a screening IHC aid for renal neoplasms driven by *TSC1/2/MTOR* and MiT family alterations (TFE3 and TFEB).^25^, ^26^ Indeed, GPNMB showed strong and diffuse staining in both tumoral components, which also highlights their commonality; of note, GPNMB was consistently negative in the fibromyomatous stroma in all cases. All three TSC patients in the current study had an altered *TSC1*, but all lacked *VHL* mutations, loss of heterozygosity at 3p or other alterations, including *ELOC* and monosomy 8.

A growing body of evidence suggests that RCC with HB‐like features belongs to the spectrum of RCC FMS with TSC/MTOR alterations. For example, a recent study by Kojima *et al*. described clinicopathologic and molecular features of RCCs exhibiting HB‐like features, which were different from clear cell RCC.^16^ They reported four sporadic cases (listed as cases 10–13 in Table 3) and found that 2/4 cases had *MTOR* mutations, and all lacked *VHL* alterations on comprehensive molecular evaluation that included DNA sequencing, 3p loss of heterozygosity analysis and methylation analysis of the VHL promoter region.^16^ The four sporadic tumours from their study, in our view, are strikingly similar to the tumours in TSC patients included in the current series. Kojima *et al*. found that all tumours had a thick peripheral fibromuscular ‘capsule’, which in 2/4 tumours extended into the tumour.^16^ All tumours had areas of ‘clear cells’ and ‘HB‐like areas’ that were intermingled with an indistinct transition between the two. As in the current study, inhibin‐α and S100 were typically expressed in the HB areas, which were CK7 negative, while the clear cell areas were CK7 positive (30%–100%), but were either completely negative for inhibin‐α and S100 or only minimally positive (<5%–10%).^16^

**Table 3:** Clinical and pathological features of tumours in TSC patients from this study (case 1–3), and data from similar sporadic tumours reported in the literature (case 4–15)

Case	Age/sex	Mutation	F/U	Size (cm)	T‐stage	WHO grade	HB %
1	19 F	Germline: Inherited copy number variant, loss of 6 genes including *TSC1*	12 mo, NED	3.5	pT1a	G1 (focally G2)	70%
2	27 F	Germline & tumour (somatic): De novo *TSC1* frameshift resulting in stop codon truncation. c.965dupT (p.Met322Ilefs*19)	10 mo, NED	1–2.1 (× 3 left) 0.4–1.3 (× 3 right)	pT1a	G2 (focally G3)	10%–100% (left) 20%–90% (right)
3	17 F	Germline: De novo *TSC1* two bp deletion: frameshift. c.1781_1782del In tumour (somatic): *TSC1* pVal594GlyfsTer11 and *TSC1* pLys890Ter	21 y, NED	2.5	pT1a	G2	5%
4 (18)	75 F	Not performed	6 mo, NED	1.7 × 1.5	NA	G2 (focally G3)	30%
5 (18)	59 M	Not performed	20 mo, NED	5.7 × 5.5	NA	G1	100%
6 (19)	49 M	Not performed	NA	3.5	NA	G1	70%
7 (19)	32 F	Not performed	NA	3.2	NA	G1	60%
8 (20)	69 M	Not performed	NA	4 × 4 × 3	NA	NR	Extensive
9 (23)	38 F	In tumour (somatic): *TSC2* c.T311C:p. L104P *SETD2* c.C721G:p.P241A	18 mo, NED	2 × 1 × 0.7	NA	G1‐G2	40%
10 (16)	78 M	In tumour (somatic): *MTOR* c.7280 T>G	19 mo, NED	1.3	pT1a	G2	40%
11 (16)	65 M	*ELOC*, *MTOR*, both reported normal*	11 mo, NED	1.8	pT1a	G2	90%
12 (16)	70 M	*ELOC*,*MTOR,* both reported normal*	10 mo, NED	1.5	pT1a	G1	40%
13 (16)	87 F	In tumour (somatic): *MTOR*: c.7280T>G	3 mo, NED	3.0	pT1a	G2	40%
14 (22)	30 M	No *VHL* mutation	NA	3.2	NA	NA	95%
15 (24)	28 F	No *VHL* alteration detected through sequencing, LOH or promoter methylation	7 y, NED	3.5	NA	NA	60%

Cases reported as ‘RCC with HB‐like features’ (18), ‘Clear cell RCC with HB‐like features’ (19, 20), ‘RCC with HB‐like features and leiomyomatous stroma’ (23), ‘RCC with HB‐like features’ (16), ‘RCC and extraneural HB’ (Case 1) (22), ‘Clear cell RCC and extraneuraxial HB’ (Case 9) (24).

bp, base pair; F/U, follow‐up; F, female; LOH, loss of heterozygosity; M, male; mo, months; NA, not available; NED, no evidence of disease; y, year.

* In tumours 11 and 12, *TSC1*, *TSC2*, *RHEB*, *SETD2*, *PBRM1* gene mutation status was not available.

Our English literature review of renal tumours with a distinct HB‐like component, reported either as exclusive renal HB or renal HB in combination with an epithelial (‘clear cell’) component, identified, in addition to those reported in the Kojima *et al*. study,^16^ six additional studies with a total of 8 cases (listed as 4–9 and 14–15 in Table 3)^18^, ^19^, ^20^, ^22^, ^23^, ^24^ All such tumours were sporadic (i.e., in non‐TSC patients), similar to those reported by Kojima *et al*.,^16^ and similar to RCC FMS tumours with HB‐like areas in TSC patients in the current study.^18^, ^19^, ^20^, ^22^, ^23^, ^24^ Although these previous studies used variable terminologies for such tumours (see Table 3), all such tumours had morphologic and IHC similarities, with diffuse CK7 positivity in the clear cell component. HB‐like component demonstrated inhibin‐α and frequent S100 positivity, while CAIX (cup‐shaped) and vimentin were positive in both.^16^, ^18^, ^19^, ^22^, ^23^, ^24^ Of note, S100 was reported as negative of variable in the HB areas (focal, <5%–30%) in some published studies.^16^, ^18^ Additionally, CD10 and PAX8 were also variably expressed in some studies with less intense, focal or negative staining in the HB‐like component.^16^, ^18^, ^20^, ^23^, ^24^ Variability in the intensity of the IHC staining may also be related to the differences in protocols or antibodies used in various studies.

In one case report, *TSC2* and *SETD2* mutations were also found in one RCC with HB‐like features and leiomyomatous stroma.^23^ Notably, none of the previous cases, reported either as pure renal HB or renal HB in combination with an epithelial (‘clear cell’) component, demonstrated either *VHL* alterations or clinical features associated with a VHL syndrome, as found in about 25% of typical cerebellar HBs.^21^, ^28^ Interestingly, in a series of 22 peripheral HBs that included 3 arising in the kidneys, one patient was reported with TSC; details of these renal HBs were, however, not provided.^17^

The relationship of RCC FMS (or RCC with leiomyomatous stroma) and renal HB remains not fully elucidated. This may be because renal HB is an exceedingly rare tumour, and because the clear cell component and the stromal component may have not been recognized or documented, if limited, in cases reported as ‘renal HB’. However, in a recent study of 10 renal HBs, Wang *et al*. showed that all were characterized by recurrent mutations in the TSC/mTOR genes, which clearly distinguished renal HBs from the HBs in the CNS that are consistently associated with VHL alterations.^29^ They further suggested that renal HBs might be part of a continuum within the spectrum, where renal HB predominantly follows a mesenchymal differentiation, RCC FMS with TSC/mTOR mutations typically demonstrates epithelial differentiation, while RCC with HB‐like features encompasses both epithelial and mesenchymal characteristics.^29^ Thus, they suggested that RCC with HB‐like features may occupy a unique position within this disease spectrum, potentially bridging the gap between the two distinct differentiation pathways.^29^

Alternatively, HB‐like areas may not have been recognized or noted, particularly if limited, in published series of RCC FMS with TSC/MTOR alterations. For example, in our view, a recent study by Tjota *et al*.^30^ of 12 *TSC/MTOR* mutated RCC with leiomyomatous stroma (LMS), illustrated HB‐like areas (in their Figures 2A,B, 2D, 3A, 3C,D) that lacked or only focally expressed CK7 (illustrated in their Figures 2F and 3G, and in cases 1, 6 and 7 listed in Table 2). The HB‐like areas were, however, not explicitly mentioned as such,^30^ and IHC evaluation for inhibin‐α and S100 was not done, which could have been helpful in characterizing them as HB‐like areas. HB‐like areas have also not been explicitly reported in other RCC FMS studies, in which such tumours were molecularly characterized by *TSC1*, *TSC2* or *MTOR* mutations, either in a sporadic setting^11^, ^12^ or in patients with TSC.^14^ Recognizing such HB‐like areas in RCC FMS may also assist with the correct diagnosis, as such areas show solid growth and IHC profile with inhibin‐α and S100 positivity, while CK7 is typically negative. In addition, GPNMB expression in tumours with clear cells would be suggestive of associated *TSC/MTOR* or *TFE3/TFEB* alterations, as demonstrated in this series and other reports.^25^, ^26^

RCC FMS is a distinct entity from clear cell RCC, clear cell (tubulo)papillary tumour and *ELOC*‐mutated RCC, as detailed in Table 4. Although all three entities may show variable presence of fibromyomatous stroma, they also demonstrate morphologic, IHC and molecular differences from RCC FMS with *TSC/MTOR* mutations. The distinction between RCC FMS and clear cell RCC is of particular importance clinically because RCC FMS with *TSC/MTOR* mutations generally has an indolent behaviour, although in rare instances it may exhibit metastatic spread.^31^ This is in contrast to the potentially aggressive behaviour of clear cell RCC. Although the distinction between RCC FMS and clear cell RCC with prominent stromal component may be challenging, especially on biopsy with limited tissue available, the morphologic clues, such as variable papillary architecture and branching tubules, diffuse CK7 and GPNMB reactivity on IHC, and *TSC/MTOR* mutations in the absence of *VHL* alterations, may aid in establishing a diagnosis of RCC FMS with TSC/MTOR alterations. Although clear cell (tubulo)papillary tumour also shows diffuse CK7 reactivity^32^, ^33^ and variable presence of fibromyomatous stroma, it has predominantly tubular, non‐branching architecture, delicate or abortive papillary structures, low‐grade, abluminal nuclei and lacks a distinct molecular signature.^32^, ^33^ *ELOC* (formerly *TCEB1*)‐mutated RCC harbours mutations in the *ELOC* (*TCEB1*) gene at 8q21.11^34^ and some cases had monosomy 8.^35^, ^36^ Although *ELOC*‐mutated RCC has been introduced as a novel entity in the 2022 WHO classification of tumours,^34^ *ELOC*‐mutated RCC exhibits a morphology and IHC profile that largely overlap with RCC FMS with *TSC/MTOR* mutations, and their distinction requires molecular evaluation, aided by IHC for GPNMB (positive in RCC FMS with *TSC/MTOR* mutations vs. negative in *ELOC*‐mutated RCC).^26^ To our knowledge, *ELOC*‐mutated RCC has not been reported or illustrated to exhibit HB‐like areas, and no RCC FMS with HB‐like morphology has been found to have *ELOC* mutations. Therefore, the recognition of morphologies with corresponding molecular findings is important in the current practice because the diagnosis may inform the prognosis and the clinical management, may identify possible targeted therapies (for example, with MTOR inhibitors) and may help identify hereditary or syndromic associations.

**Table 4:** Clinicopathologic, immunohistochemical and molecular features of central nervous system hemangioblastoma, renal hemangioblastoma and renal cell carcinoma with fibromyomatous stroma with hemangioblastoma‐like areas

	CNS HB	Renal HB	RCC FMS with HB‐like areas
Clinical features	~70% sporadic, 60%–80% with VHL Sy develop CNS HB	No known predispositions	Sporadic or associated with TSC
Gross findings	Well‐circumscribed, pseudo‐encapsulated cystic mass with a variegated nodule or (less likely) entirely solid mural nodule	Well‐circumscribed, solid, red brown, grey, to yellow	Well‐circumscribed, tan/yellow/grey‐white to red, surrounded by solid fibromuscular band. May be unifocal or multifocal (if in TSC)
Histology	Two components: (1) large multi‐vacuolated, lipidized stromal cells often with pleomorphic or enlarged nuclei; (2) intricate capillary network	Similar to CNS HB. Thick fibromuscular stroma in many cases. May have cytoplasmic eosinophilic globules	Two components: (1) clear cytoplasm, arranged in nests, branching tubules or papillae; (2) peripheral fibromyomatous stroma may intersect the tumour. HB‐like areas similar to CNS HB
IHC	*Positive*: inhibin‐α, NSE, S100, D2‐40 and brachyury (cytoplasmic), CAIX (membranous) *Negative*: keratins (rarely focally positive), CK7, EMA, CD10, PAX8, vimentin	*Positive*: inhibin‐α, S100, PAX8, NSE and vimentin. Focal pos: keratins, CD10, CAIX and EMA *Negative*: CK7, HMB45 and Melan‐A	*Positive*: CK7 and AE1/AE3 *Negative*: inhibin‐α, S100 *Positive*: inhibin‐α, S100 *Negative*: CK7 and AE1/AE3 (or focally positive) Both areas positive: GPNMB, CD10, CAIX, vimentin
Molecular	*VHL* gene alterations, including allelic losses, inactivation or mutation	No known molecular alterations, no *VHL* mutations reported	*TSC1/2*, *MTOR*, *SETD2* (one case) Negative for *VHL* mutations
Prognosis	Excellent prognosis after complete excision	No tumours reported to recur or metastasize	Excellent prognosis (rare metastases reported in RCC FMS without HB areas)

CNS, central nervous system; HB, hemangioblastoma; RCC FMS, renal cell carcinoma with fibromyomatous stroma; TSC, tuberous sclerosis complex; VHL, Von Hippel Lindau syndrome.

In this series of TSC patients, we provide further insight into the morphologic spectrum of TSC‐associated RCC FMS, and we highlight the underrecognized occurrence of HB‐like areas in RCC FMS in TSC patients. In our view, the commonalities between RCC FMS and RCC FMS with HB‐like features support the conclusion that HB‐like features are part of the spectrum of RCC FMS with *TSC/MTOR* alterations. We hope that future studies that incorporate clinical, morphologic, IHC and molecular evaluations will confirm that RCC FMS with *TSC/MTOR* mutations is a distinct entity that may exhibit variable HB‐like areas, both in sporadic and in TSC settings.

## Author contributions

K. Trpkov designed the study. K. Baranova, L. Lockau, L. Ninivirta and J. A. Houpt analysed the data and drafted the manuscript under the supervision of J. A. Gomez and M. Gabril. K. Baranova and A. A. House supervised D. Arnold in data analysis and preparation of the tables. A. A. House and S. Pautler assisted with patient identification, patient consent and data analysis. F. Siadat, K. Baranova and K. Trpkov prepared the figures. M. Moussa, R. Saleeb, H. Chen, J. A. Gomez, M. Gabril, A. A. House, S. Pautler, F. Siadat, A. Yilmaz, A. Box, F. Zemp and D. Mahoney performed data collection, editing and critical feedback on the manuscript.

## Funding information

Authors received no financial support for the research, authorship and/or publication of this article.

## Conflict of interest

The authors declare no potential conflicts of interest with respect to the research, authorship and/or publication of this article.

## Supporting information


**Figure S1.** Patient 1—initial biopsy. **A**–**C**. Initial biopsy performed in patient 1, showed only HB‐like areas with solid growth composed of eosinophilic to focally finely vacuolated, epithelioid and stromal cells. (**D**) CK7 was negative. (**E**, **F**) Inhibin‐α (**E**) and S100 (**F**) were uniformly positive.

Data S1.

## Data Availability

The data that support the findings of this study are available on request from the corresponding author.
