# Supplementary figures and images for: Renal cell carcinoma with fibromyomatous stroma (RCC FMS) and with hemangioblastoma‐like areas is part of the RCC FMS spectrum in patients with tuberous sclerosis complex
Source: Histopathology. 2025 Jul 1;87(5):687–99. doi: 10.1111/his.15505 (PMC12522018; doi:10.1111/his.15505)

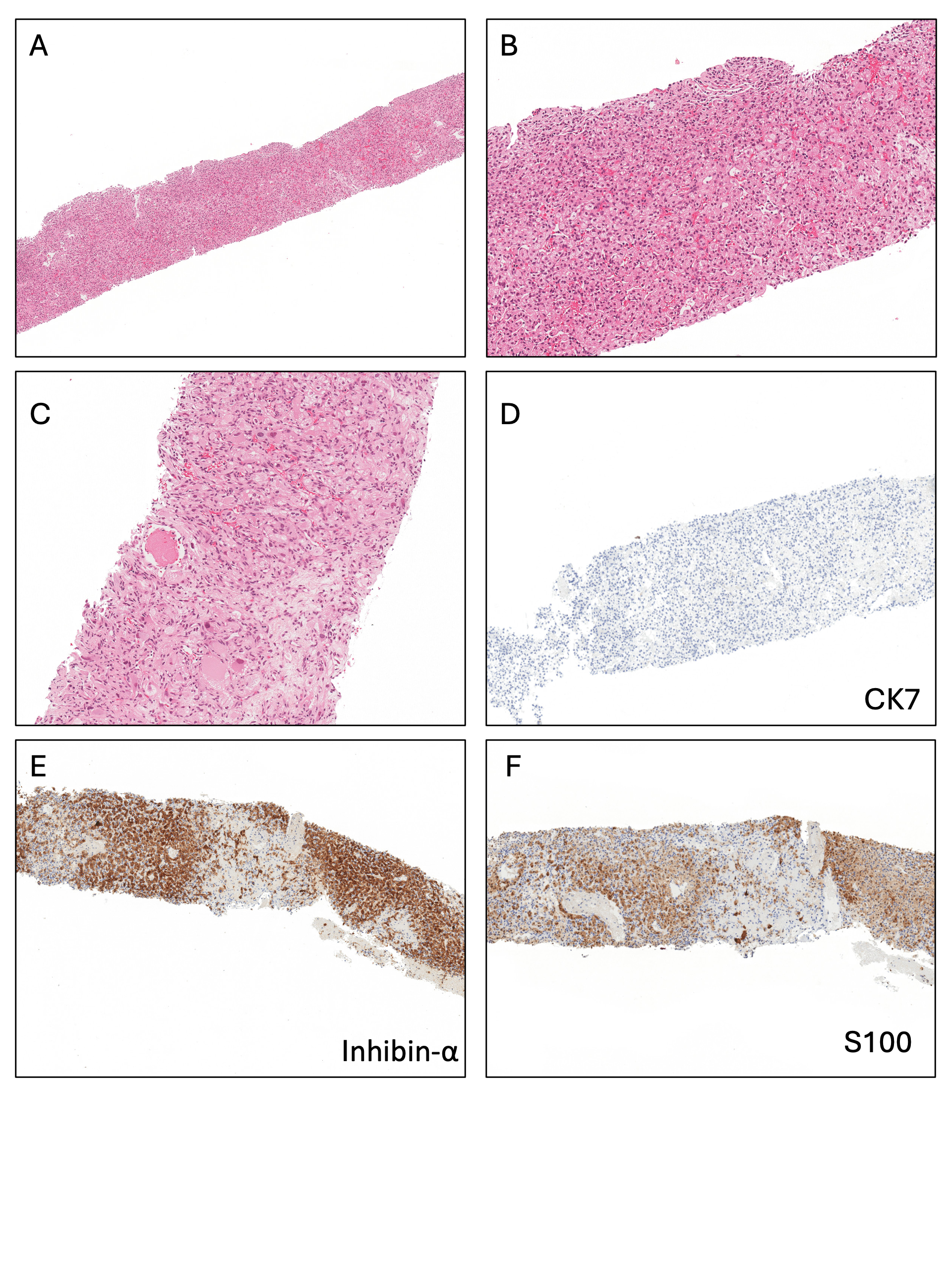

Supplement: Supplementary file 1 — Figure S1. Patient 1—initial biopsy. A–C. Initial biopsy performed in patient 1, showed only HB‐like areas with solid growth composed of eosinophilic to focally finely vacuolated, epithelioid and stromal cells. (D) CK7 was negative. (E, F) Inhibin‐α (E) and S100 (F) were uniformly positive. [file HIS-87-687-s001.tiff]
