# Supplementary material for: Renal cell carcinoma with fibromyomatous stroma (RCC FMS) and with hemangioblastoma‐like areas is part of the RCC FMS spectrum in patients with tuberous sclerosis complex
Source: Histopathology. 2025 Jul 1;87(5):687–99. doi: 10.1111/his.15505 (PMC12522018; doi:10.1111/his.15505)
Supplement: Supplementary file 2 — Data S1. [file HIS-87-687-s002.docx]

**Supplemental Table 1**. Additional IHC comparing the hemangioblastoma-like areas (HB-like) to areas of renal cell carcinoma with fibromyomatous stroma (RCC FMS).

|  | Patient 1 | | Patient 2 | | Patient 3 | |
| --- | --- | --- | --- | --- | --- | --- |
|  | **HB-like** | **RCC FMS** | **HB-like** | **RCC FMS** | **HB-like** | **RCC FMS** |
| Cathepsin | - (b) | ND | + (f) | - | ND | ND |
| CD117 | - | - | - | - | ND | ND |
| CD31/CD34 | + (v) | + (v) | + (v) | + (v) | ND | ND |
| CK20 | - | - | - | - | ND | ND |
| CK8/18 | ND | ND | ND | ND | - | + |
| CK5/6 | ND | ND | - | - | - | + (f) |
| CKPAN* | + (f) | + | - | + | - | + |
| D2-40 | - | - | ND | ND | ND | ND |
| Desmin | - | + (f, stroma) | - | + (f, stroma) | ND | ND |
| EMA | + (f) | + | ND | ND | ND | ND |
| Glut-1 | + | + | ND | ND | ND | ND |
| HMB-45 | - | - | - | - | ND | ND |
| HMWCK | - (b) | ND | ND | ND | - | + |
| MDM2 | - (b) | ND | ND | ND | ND | ND |
| MelanA | - | - | - | - | ND | ND |
| NSE | + | - | ND | ND | ND | ND |
| PAX8 | + (f) | + | ND | ND | + (f) | + |
| SDHB | + (r) | + (r) | + (r) | + (r) | ND | ND |
| SMA | + (stroma) | + (stroma) | + (stroma) | + (stroma) | ND | ND |
| Synaptophysin | - | - | ND | ND | ND | ND |
| TFE3 | - | - | - | - | ND | ND |
| WT1 | - | - | ND | ND | - | - |
| AMACR | ND | ND | - | - | ND | ND |
| GATA | ND | ND | - | - | ND | ND |
| Ki67 | ND | ND | <1% | <1% | ND | ND |

Abbreviations: f: focal; v: vessels, ND: not done; r: retained nuclear staining; b: done on biopsy. CKPAN: cocktail of AE1/AE3 and Cam 5.2.

**Supplemental NGS Methodology**

OVERVIEW: DNA extraction is performed using the MaxMAX protocols in the KingFisher Apex purification system. The assay utilizes pools of primers to target the full coding sequence or hot-spot regions of 130 genes, 76 microsatellite loci, and several single nucleotide polymorphism (SNP) loci distributed across all the chromosomes. The template preparation of the Ion AmpliSeq-based libraries is performed in the Ion Chef System and massively parallel sequencing is performed in the Ion GeneStudio S5 Systems. Base calling is generated by the Torrent Suite Software (version 5.18.1). Variant calling, Oncomine variant annotations, and tumor mutational burden (TMB) status are generated by Ion Reporter software (version 5.16) with alignment to the reference human genome GRCh37/hg19. Copy number alterations (CNA) are evaluated using OncoCNV software (version 6.9). Loss-of-heterozygosity (LOH) is evaluated using SNPitty software. Microsatellite instability (MSI) analysis is performed using MSICaII plugin (version 4.2).

INTERPRETATION: Tier I, II, and III variants (per AMP/ASCO/CAP guidelines) present at 5%-100% variant allele fraction (VAF) are reportable. Tier I variants have strong clinical significance with level A and B evidence. Tier II variants have potential clinical significance (level C and D evidence). Tier III variants are variants of unknown clinical significance. Functional assessment of the variants (oncogenicity) follows the ClinGen/CGC/VICC criteria. MSI-High status is defined by an established MSICaII score and/or the presence of mutations in the mismatch repair genes. This assay may be used as a screening test for tumors with TMB-High status where the TMB score is expected to be equal or superior to 10 mutations per megabase (mut/Mb); however, confirmation of the TMB-High status by an orthogonal method is encouraged if clinically indicated. All genomic coordinates are given with respect to GRCh37 (hg19). For cDNA and protein coordinates, reference transcripts are listed in the report.

REGIONS COVERED: The Cancer Biomarker Comprehensive DNA panel is a pan-solid tumor assay validated to detect single nucleotide variants (SNVs), small insertions or deletions (indels), amplifications, and homozygous-type deletions in the following 130 genes: ACVR1, AKT1, AKT3, ALK, APC, AR, ARAF, ARHGAP35, ARID1A, ATM, ATRX, BAP1, BARD1, BCOR, BRAF, BRCA1, BRCA2, BRIP1, CCND1, CCNE1, CDC42, CDH1, CDK12, CDK4, CDK6, CDKN2A, CDKN2B, CHEK2, CIC, CTNNB1, DICER1, EGFR, EIF1AX, ELOC, EPCAM, ERBB2, ERBB3, ERBB4, ESR1, EZH1, EZH2, FBXW7, FGFR1, FGFR2, FGFR3, FGFR4, FH, FLCN, FOXL2, FUBP1, GNA11, GNAQ, GNAS, H3F3A, H3F3B, HIST1H3B, HIST1H3C, HNF1A, HOXB13, HRAS, IDH1, IDH2, KEAP1, KLF4, KIT, KRAS, MAP2K1, MAP2K2, MAP2K4, MAPK1, MDM2, MDM4, MEN1, MET, MLH1, MSH2, MSH6, MTOR, MYC, MYCN, MYD88, MYOD1, NBN, NF1, NF2, /\/RAS, NTRK1, NTRK2, NTRK3, PALB2, PBRM1, PDGFRA, PDGFRB, PIK3CA, PIK3CB, PIK3R1, PLEKHS1, PMS2, POLE, POLR2A, PPP2R1A, PRKCA, PRKD1, PTEN, RAD51C, RAD51D, RAC1, RAF1, RB1, RET, ROS1, SDHA, SDHB, SDHC, SDHD, SETD2, SMAD4, SMARCA4, SMARCB1, SMO, SPOP, STK11, TERT, TFEB, TP53, TRAF7, TSC1, TSC2, TSHR, and VHL. Loss-of-heterozygosity (LOH) with corresponding copy number status (gain, loss, or copy-neutral) is assessed in all chromosome arms and reported when clinically valuable. The acceptable minimum depth of coverage is 100X and the mean depth is 1000X. Certain regions of some genes are not reported due to low coverage (a list of these regions is available upon request).

TEST LIMITATIONS: Small insertions or deletions occurring in a homopolymer sequence of 8 or more mononucleotide repeats are not detected. This assay requires a minimum of 10% tumor cellularity for the accurate detection of SNVs and small indels; 20% tumor cellularity for chromosomal LOH, MSI-High status, and TMB-High status, and 30%-50% tumor cellularity for homozygous-type deletions. For an accurate detection of gene amplification, the tumor cellularity requirement is variable and ranges from 10% tumor cellularity (if the number of copies per tumor cell is equal to or exceeds 32) to 100% tumor cellularity (if the number of copies per tumor cell is equal to or exceeds 5). A reliable distinction between variants located in the PMS2 gene and/or pseudogene(s) cannot be made due to the existence of homologous sequences and the confirmatory testing to determine the variant location cannot be performed on tissue specimens.

PERFORMANCE METRICS: When the minimum specimen requirements are met, the positive percentage agreement is 99% for SNV, 96% for small indels (verified for indels of less than 40 bp in size), 100% for copy number alterations, 100% for LOH in chromosomal arms, and 100% for MSI-High detection, and the negative percentage agreement is 100% respectively. The evaluation of TMB-High analysis in a small validation cohort yielded 100% positive percent agreement and 98% negative percentage agreement. Specimens with increased deamination artifacts or expected TMB values close to the 10 mut/Mb cut-off may be reported as indeterminate for TMB-High status. Rare polymorphisms may be present and could lead to false negative or false positive results. A negative (wild-type) result does not rule out the presence of a molecular alteration below the limits of detection of the assay.

References:

[1] Li M, et al. Standards and Guidelines for the Interpretation and Reporting of Sequence Variants in Cancer: A Joint Consensus Recommendation of the Association for Molecular Pathology, American Society of Clinical Oncology, and College of American Pathologists. J Mol Diagn. 2017 Jan;19(1):4-23.

[2] Horak P, et al. Standards for the classification of pathogenicity of somatic variants in cancer (oncogenicity): Joint recommendations of Clinical Genome Resource (ClinGen), Cancer Genomics Consortium (CGC), and Variant Interpretation for Cancer Consortium (VICC). Genet Med. 2022 May;24(5):986-998.
